# Exosomes isolation and identification from equine mesenchymal stem cells

**DOI:** 10.1186/s12917-019-1789-9

**Published:** 2019-01-28

**Authors:** Michele Christian Klymiuk, Natalie Balz, Mohamed I. Elashry, Manuela Heimann, Sabine Wenisch, Stefan Arnhold

**Affiliations:** 1 Institute of Veterinary-Anatomy, -Histology and -Embryology, Faculty of Veterinary Medicine, Justus-Liebig-University Giessen, Frankfurter Strasse 98, Giessen, 35392 Germany; 2 Clinic of Small Animals, c/o Institute of Veterinary-Anatomy, -Histology and -Embryology, Faculty of Veterinary Medicine, Justus-Liebig-University Giessen, Frankfurter Strasse 98, Giessen, 35392 Germany; 3 Anatomy and Embryology Department, Faculty of Veterinary Medicine, University of Mansoura, Mansoura, 35516 Egypt

**Keywords:** Exosomes, Equine mesenchymal stem cells, Stem cells, Nanoparticle tracking analysis

## Abstract

**Background:**

Mesenchymal stem cells are used for different therapeutic approaches, e.g. for osteoarthritis, lesions of the tendon as well as for bone defects. Current research on the mechanism of stem cells on the repair of damaged tissue suggest an important role of a cell-to-cell communication through secreted extracellular vesicles, mainly represented by exosomes. To enhance the scarce knowledge on the functional role of exosomes we compared as a first step different techniques to isolate and identify exosomes from the supernatant of equine adipose derived mesenchymal stem cells for further characterization and usage in functional assays.

**Results:**

It was possible to obtain exosomes secreted from equine adipose derived mesenchymal stem cells with three common techniques: a stepwise ultracentrifugation at 100.000 g, an ultrafiltration with 3 kDa exclusion membranes and a charge-based precipitation method. The mean sizes and amounts of exosomes isolated with the different techniques were measured by the nanoparticle tracking analysis. The diameter ranged between 116.2 nm (ultracentrifugation), 453.1 nm (precipitation) and 178.7 nm (ultrafiltration), the counts of particles / ml ranged between 9.6 × 10^8^ (ultracentrifugation), 2.02 × 10^9^ (precipitation) and 52.5 × 10^9^ (ultrafiltration). Relevant marker for exosomes, tetraspanins CD9, CD63 and CD81 were detectable by immunofluorescence staining of the investigated exosomes secreting mesenchymal stem cells. In addition, transmission electron microscopy and immunogold labeling with CD9 and CD90 was performed to display the morphological shape of exosomes and existence of marker relevant for exosomes (CD9) and mesenchymal stem cells (CD90). Western blot analysis of CD9 and CD90 of exosomes ensured the specificity of the rare available respectively cross reacting antibodies against equine antigens.

**Conclusion:**

Exosomes generated by equine mesenchymal stem cells can be obtained by ultrafiltration and ultracentrifugation in an equal quality for in vitro experiments. Especially for later therapeutic usage we recommend ultrafiltration due to a higher concentration without aggregation of extracellular vesicles in comparison to exosomes obtained by ultracentrifugation.

## Background

Mesenchymal stem cells (MSC), which can be isolated from different tissues such as adipose tissue, bone marrow and other tissues such as amniotic fluid and umbilical cord, can be propagated for several passages and show a differentiation potential into various cells types and lineages e.g. adipose, osteogenic and chondrogenic lineages [1, 2]. Because of this multipotent differentiation capacity MSC have been thoroughly investigated for their therapeutic potential for various diseases. In veterinary medicine a therapeutic usage was preferentially suggested for orthopedic disorders such as tendon lesions, osteoarthritis as well as bone defects [3]. The beneficial effect was always thought to be related to differentiation of stem cells into the desired cell types of the lesioned host tissue. However, as MSC also have been shown to have an interaction with immune cells [4–6] and can even be beneficial in the treatment of graft versus host disease [7] an immunomodulatory effect is evident. Because of this immunomodulatory potential it has been proposed that the therapeutic potential of MSC is generally based on a paracrine rather than a cell dependent manner [8]. Thus, for several diseases it has been shown that the application of conditioned media of MSC is potent enough to reduce various disease states [9, 10]. This therapeutic action can most likely be attributed to the release of cytokines into the culture medium qualifying MSC as bioreactors synthesizing the appropriate factors relevant for tissue regeneration [3]. In recent years it has become more and more evident, that the therapeutic active components of MSC are not only soluble factors but additionally vesicular structures, which could be isolated from MSC supernatants by ultracentrifugation [11]. Among the group of microvesicles are vesicles, which are released into the extracellular environment of cells. Thus, they are termed as extracellular vesicles [12, 13]. Further in depth studies revealed that extracellular vesicles secreted from MSC include microvesicles with a diameter of 0.1–1 μm and exosomes (40–100 nm in diameter) [14]. It could be shown that the administration of MSC-derived exosomes may be used for a cell-free MSC therapy [15] by transporting paracrine factors during angiogenesis, mediating cell-cell micro-communication, immune regulation and tissue regeneration [16, 17]. One of the advantages using exosomes as the therapeutic agents is that these extracellular vesicles can be characterized by the expression of specific marker proteins from the tetraspanin superfamily such as CD9, CD63 and CD81 [18]. These markers were commonly expressed on the membrane surface of exosomes and were important for the formation and transportation within the cell as well as for the recognition of target cells. In order to pave the way for a later clinical usage of exosomes, the aim of the presented study was to isolate exosomes from supernatants of equine adipose tissue derived stem cells (ASC) and to characterize these exosomes by immunohistochemistry, nanoparticle tracking analysis (NTA), transmission electron microscopy (TEM), immunoelectron microscopy as well as Western blot analysis.

## Results

### Detection of exosome markers in ASC

**Fig. 1:**
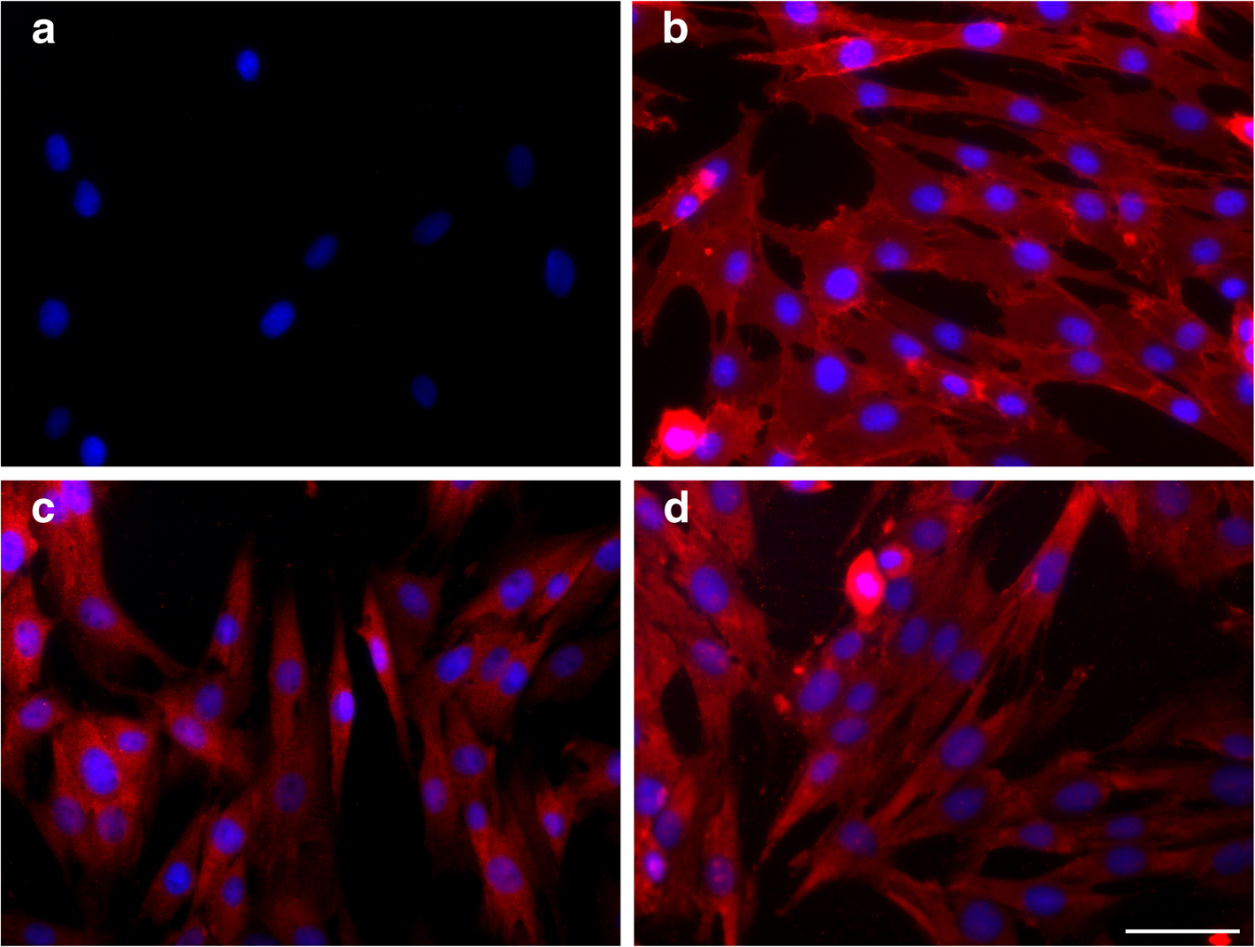
Immunofluorescence staining of equine adipose derived stem cells with CD9 (**b**), CD63 (**c**) and CD81 (**d**). Negative control with non-immune serum (**a**). Scale bar = 25 μm

In order to get a first impression whether cell-to-cell communication in stem cells involve exosomes we looked for an expression of the appropriate markers from the tetraspanin family in ASC by immunocytochemical analysis. Expression of all three tetraspanins CD8, CD63 and CD81 were observed in ASC after cultivation in cell culture dishes showing a spot like pattern in the cytoplasm (Fig. 1).

### Exosome isolation

**Fig. 2:**
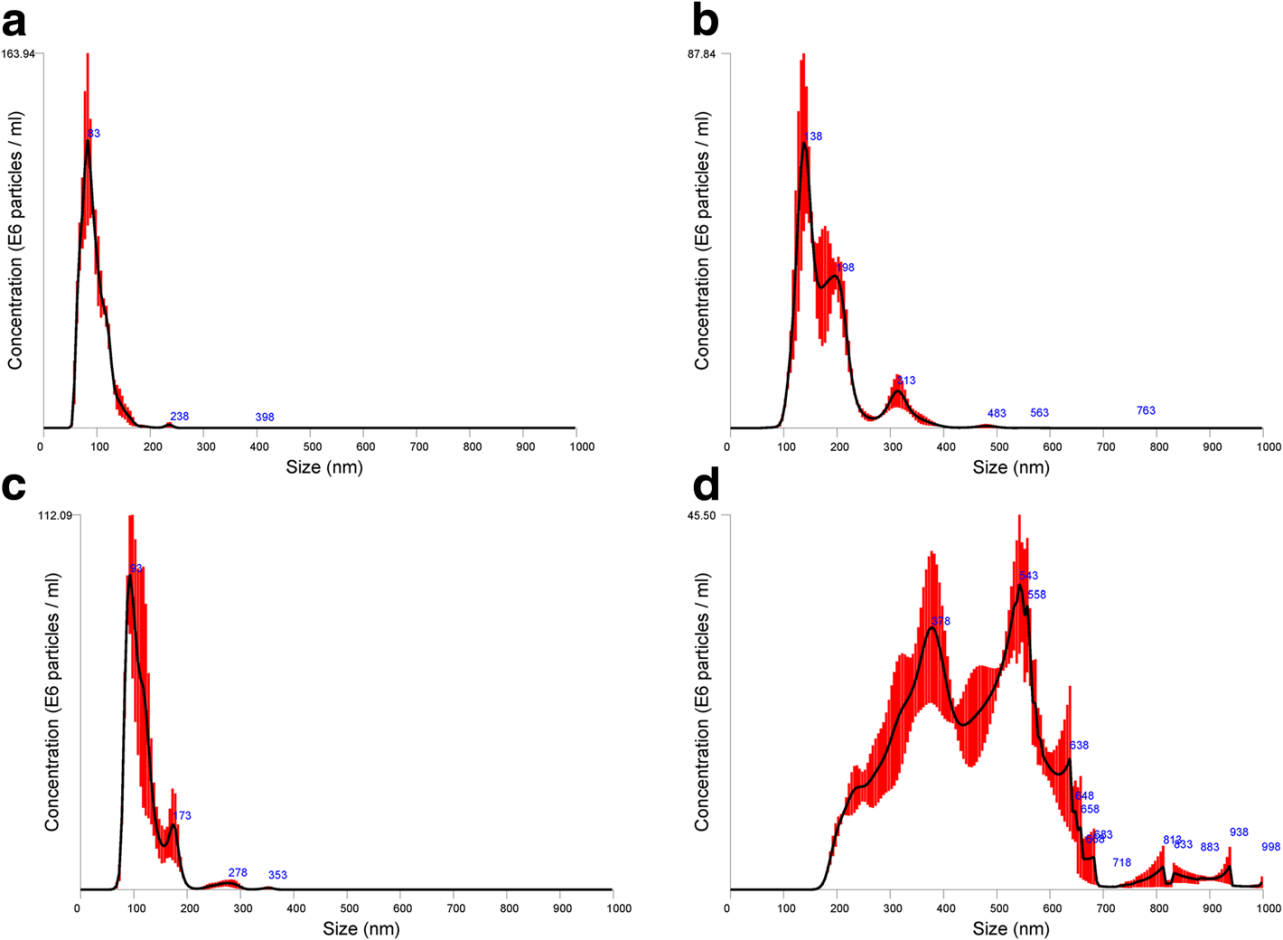
Average size and concentration after different exosome isolation techniques measured by nanoparticle tracking analysis. Native (**a**), ultrafiltration (**b**), ultracentrifugation (**c**) and precipitation (**d**) technique. The red background indicates ±1 standard error of mean of the triplicate measurement

**Fig. 3:**
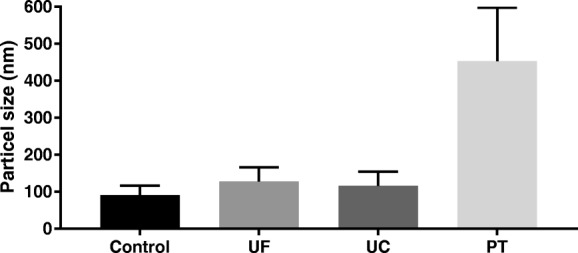
Diameter of isolated particles (exosomes). Native cell culture supernatant (Control), exosomes obtained by ultrafiltration (UF), ultracentrifugation (UC) and precipitation (PT). Error bars indicates the SD

**Fig. 4:**
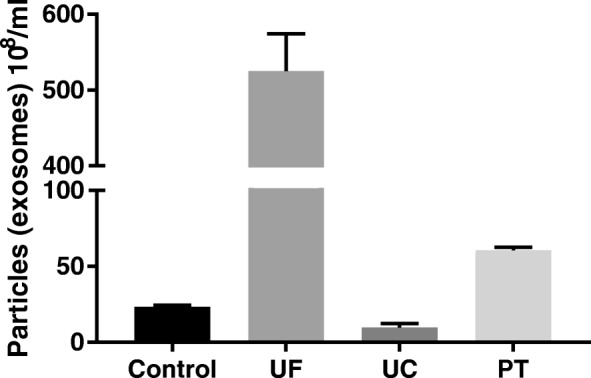
Counts of particles (exosomes). Native cell culture supernatant (Control), exosomes obtained by ultrafiltration (UF), ultracentrifugation (UC) and precipitation (PT). Error bars indicates the SD

Exosome isolation was carried out by ultracentrifugation (UC), ultrafiltration (UF) as well as by charge based precipitation (PT) involving protamine sulfate. The quantity in particles per ml and the quality in relation to the peak size distribution within the different isolation methods of exosomes were analyzed by a nanoparticle tracking analysis (Figs. 2, 3 and 4). Native exosomes from the cell culture supernatant had a peak particle size of 83–398 nm with a mean of 91.3 ± 24.7 nm (Fig. 2a and Fig. 3). After ultracentrifugation, the standard isolation technique, peak particle diameter was between 93 nm and 353 nm with a mean of 116.2 ± 38.3 nm (Fig. 2b and Fig. 3). The exosome isolation by ultrafiltration results in a peak particle diameter between 138 nm to 763 nm with a mean (± SD) at 178.7 ± 62.3 nm (Fig. 2c and Fig. 3). Exosomes isolated by the charge based precipitation method lead to an increased diameter variation due to a higher amount of coagulated exosomes, so that hardly single exosomes could be detected: the peak particle diameter ranged from 378 nm to 998 nm with a mean ± SD of 453.1 ± 144.5 nm (Fig. 2d and Fig. 3). The mean concentration of released exosomes was also calculated. By far the highest amount was obtained by ultrafiltration with a mean ± SD of 52.5 × 10^9^ ± 4.925 × 10^9^, followed by precipitation with 6.06 × 10^9^ ± 1.97 × 10^8^ exosomes per ml, respectively. Ultracentrifugation results in the lowest concentrated exosome suspension with 9.6 × 10^8^ ± 2.72 × 10^6^/ml, compared to the native concentration of 2.32 × 10^9^ ± 1.18 × 10^8^ exosomes/ml (Fig. 4).

### Detection of exosomes by transmission electron microscopy

**Fig. 5:**
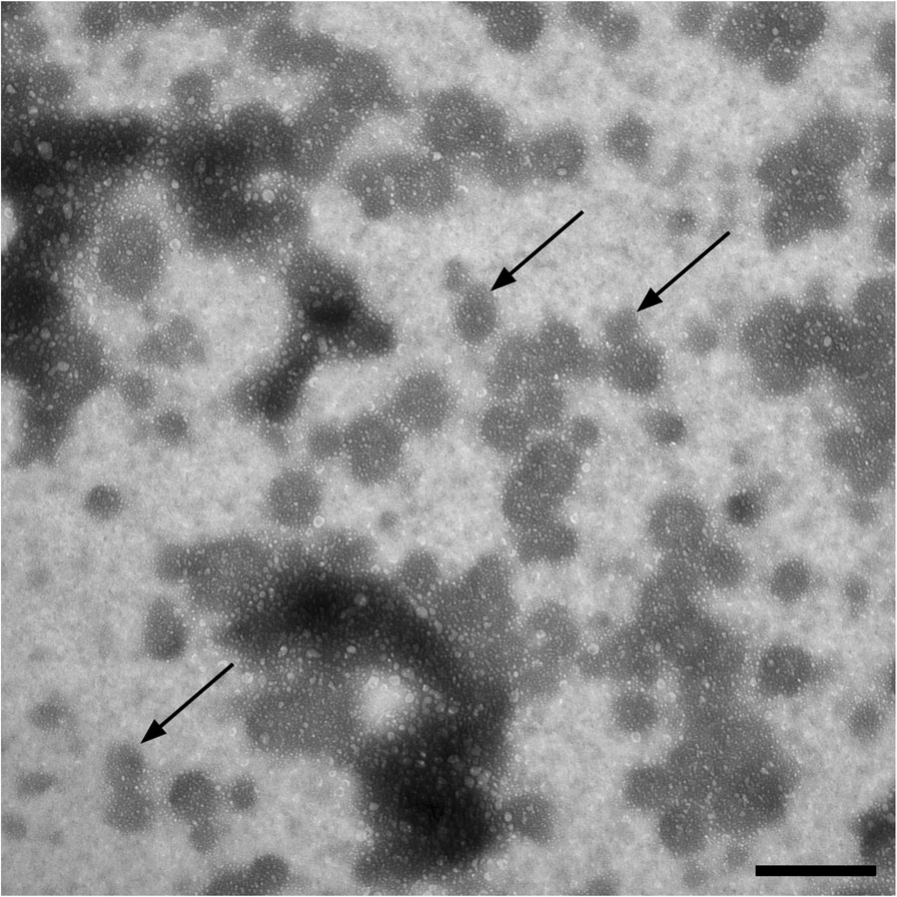
Transmission electron microscopy of exosomes. Many different sized exosomes are detectable. Scale bar = 250 nm

Morphology of isolated exosomes was investigated by transmission electron microscopy (TEM). Ultrastructural investigation of isolated exosomes revealed the expected size distribution and membrane integrity. As was already apparent using the NTA also in TEM images a heterogeneous population of exosomes was detected. Some exosomes are spherical; others are heterogeneous in shape (Fig. 5).

**Fig. 6:**
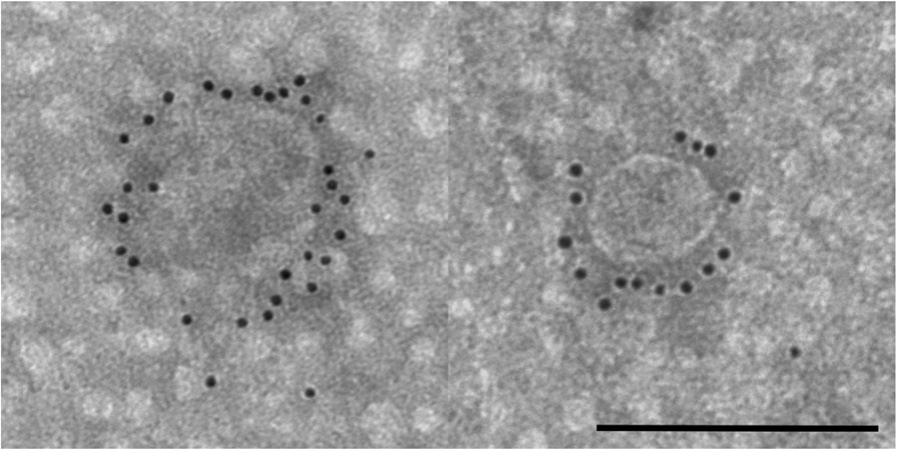
Immunogold labeled exosomes with anti CD9 antibody. Scale bar = 250 nm

**Fig. 7:**
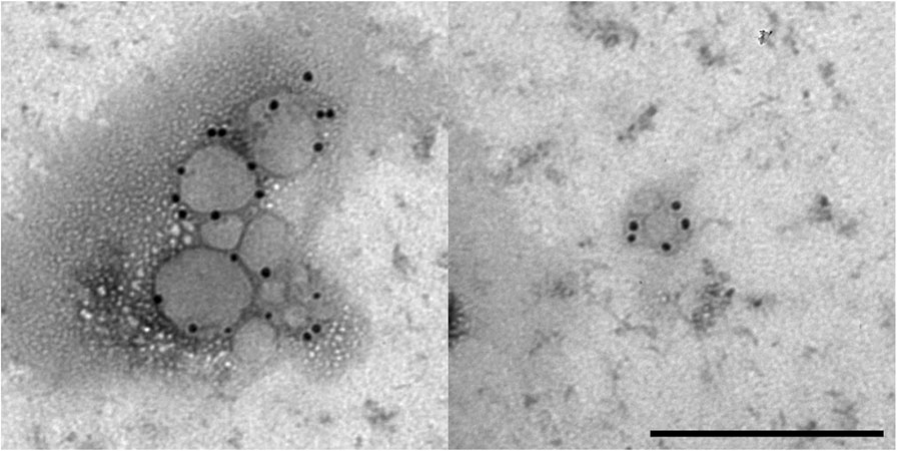
Immunogold labeled exosomes with anti CD81 antibody. Scale bar = 250 nm

**Fig. 8:**
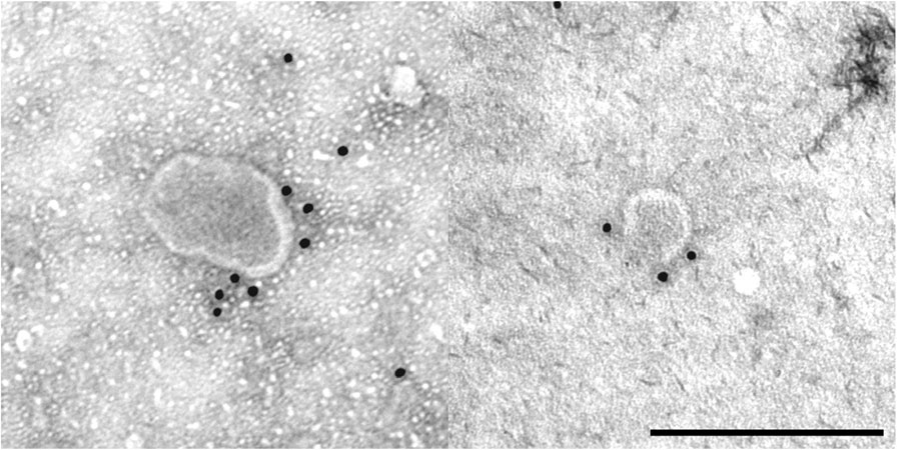
Immunogold labeled exosomes with anti CD90 antibody. Scale bar = 250 nm

The appearance of relevant exosomal and ASC markers were visualized by immunogold labeling. Black punctate regions indicate a positive staining of the tetraspanins CD9 and CD81 as well as the surface marker CD90, which is usually present in mesenchymal stem cells (Figs. 6, 7 and 8).

### Detection of stem cell and exosome markers using western blot

**Fig. 9:**
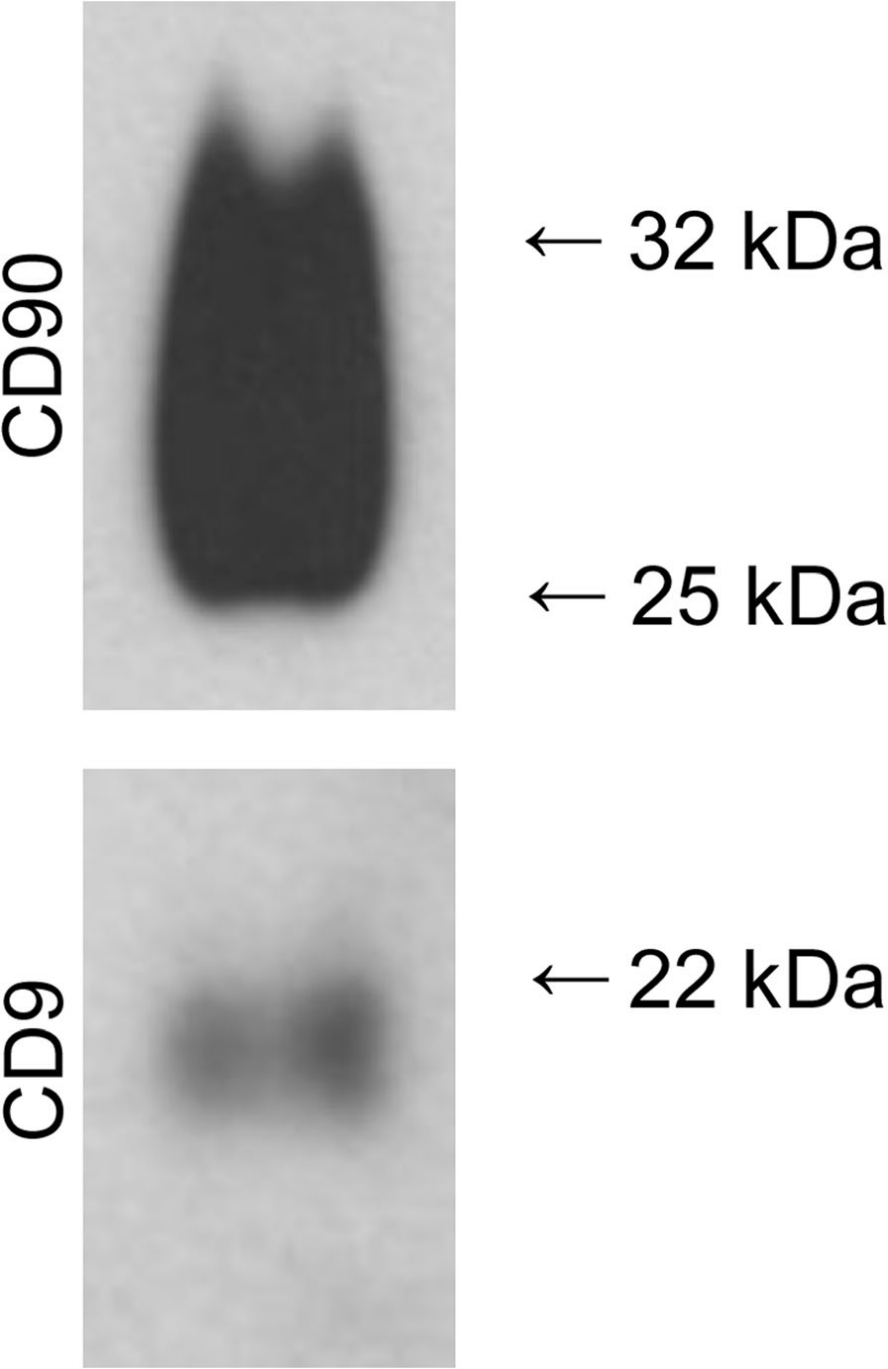
Western blot analysis of exosomes obtained by ultrafiltration. Markers CD9 and CD90 were expressed by exosomes isolated by the ultrafiltration technique

Using western blot analysis, markers for stem cells (CD90) and the tetraspanin CD9 have been detected in lysates of exosomes isolated by the ultrafiltration technique. It was not possible to detect CD9 in samples obtained from ultracentrifugation, neither from precipitation nor from native cell culture supernatants due to the low protein respectively exosome concentrations (Fig. 9).

## Discussion

Exosomes are a group of secreted vesicles, which also include membrane particles, microvesicles, exosome-like vesicles or even apoptotic bodies [19]. Exosomes originate from the endosomal membrane and were released after fusion of multivesicular bodies with the plasma membrane into the extracellular space [20–22]. Exosomes tend to be relatively homogeneous in size between 50 and 150 nm, which is within the overlapping size ranges of exosomes and microvesicles [23] are released by p53-regulated exocytosis and can be distinguished from the larger microvesicles (130–1000 nm) as well as the much larger apoptotic bodies ranging from 1 to 5 μm by size and morphology [13].

Exosomes are key players in cell-to-cell communication and immunomodulation [24]. They are from a growing interest due to the fact that exosomes also may play an important role in the healing capacities of mesenchymal stem cells [25]. In many earlier publications it could be shown that the therapy of mesenchymal stem cells in veterinary medicine could be helpful, for example in the therapy of joint and ligature diseases in the horse [26, 27]. Thus was our intention to investigate in the isolation and characterization of exosomes from equine ASCs.

Exosomes can be isolated from supernatants of various cells and tissues including stem cells by different isolation techniques such as ultracentrifugation, ultrafiltration and a charge based precipitation method involving protamine sulfate. While isolation of exosomes by ultracentrifugation is the most commonly applied method, exosome isolation by ultrafiltration may guarantee a gentler isolation and thus a higher rate of yield. The first step to proof the occurrence of exosomes in equine ASC in our study has been done immunocytochemically by the evidence of an expression of exosomal tetraspanins in cultured ASC: CD9, CD63 and CD81 staining was positive in ASC, it revealed in punctate pattern inside the cytoplasm. The expression of the analyzed tetraspanins has been specifically described for extracellular vesicles such as exosomes [28] and were suggested by the International Society of Extracellular Vesicles (ISEV) for the identification of exosomes [29].

The nanoparticle tracking analysis (NTA) allowed us to show that with all three isolation methods exosomes can be isolated from culture conditioned medium of ASC. While ultracentrifugation is the most widely used method for exosome isolation and was once called the “gold standard” for exosome preparation [30], our study indicated that very good or even better results could also be achieved by a size based isolation technique applying ultrafiltration. In comparison to the ultracentrifugation method, the highest values were slightly more heterogenic compared to the native sample of conditioned medium. However, the isolation of exosomes by ultrafiltration resulted in a 50-fold increased concentration than obtained by ultracentrifugation. In addition, ultracentrifugation was far more time consuming than ultrafiltration taking only about 30 min to concentrate 150 mL of conditioned medium compared to two ultracentrifugation steps of a duration of 90 min each. This has been similarly described for the isolation of exosomes from the supernatant of a squamous lung cancer cell line [31].

In contrast to this, the charge-based precipitation methods, relies on the negative charge of extracellular vesicles, which can be recovered by a positively charged protamine in a polymeric matrix [32]. The charge based isolation gave a very good yield in exosome retrieval, too. However, very often next to single exosomes using the precipitation method there is a tendency of exosomes to aggregate, resulting in larger particles as can be analyzed using the NTA.

This is in contrast to observations by Deregibus et al. 2016 [32] who observed similar to our data an efficient recovery of exosomes by the protamine precipitation compared to ultracentrifugation with a size range of particles between 35 to 95 nm depending on the source of EVs. Interestingly, they did not have any evidence for an aggregation of vesicles.

NTA results can be confirmed by transmission electron microscopy. Using electron microscopy, the morphology of exosomes can be studied in detail. TEM images revealed that ASC derived exosomes have a cup-shaped appearance which can be detected as membrane-bound vesicles. The morphology and size were consistent with previous reports [33]. Furthermore, immunogold TEM images reveal the presence of the tetraspanin marker CD9 and CD81 as well as the labelling with the stem cell marker CD90, indicating that the isolated exosomes are truly stem cell derived.

Overall the availability of suitable antibodies is a remarkable problem for the identification and characterization of equine exosomes. Suitable antibodies for the equine species are extremely scarce and thus we tested a series of antibodies for a possible cross reactivity on equine antigens. In addition, not all antibodies worked with the different techniques we investigated. For this reason, we could not test more antigens that would normally be common for the characterization of exosomes.

As exosomes hold numerous potential applications in diagnostics and therapeutics robust exosome isolation procedures are the cornerstone for obtaining meaningful results.

Therefore, we can show for the first time to our knowledge in veterinary medicine, that exosomes can be easily isolated from the supernatants of equine adipose tissue derived stem cells.

## Conclusions

With our experiments we can unequivocally demonstrate that exosomes can be efficiently isolated from stem cell conditioned medium from equine ASC. Isolation can be carried out by application of different isolation procedures from which ultrafiltration is a time and cost-effective alternative. Based on these aspects, we propose that ASC as supportive cells target with their secreted extracellular vesicles intrinsic processes to revitalize homeostasis in damaged tissue and thus initiate tissue regeneration. These tissue related features provide a perspective for the therapeutic efficacy of ASC and their secreted exosomes in a wide spectrum of diseases also in veterinary medicine. Further experiments have to actually prove the therapeutic potential of ASC derived exosomes in veterinary-related lesions and diseases.

## Methods

### Isolation and culture of equine adipose tissue derived mesenchymal stem cells

Subcutaneous adipose tissue was collected from the region above the dorsal gluteal muscle from 3 mixed breed male or female horses (aged, mean ± SD, 14.33 ± 4.78 years) as previously described by Raabe et al. 2010 [34]. All samples were obtained from slaughtered horses at the local abattoir. The adipose tissue was diced into small pieces and digested for 40–60 min at 37 °C with 0.1% collagenase type I (Biochrom AG, Germany) in phosphate-buffered saline (PBS, Thermo Fisher Scientifc, Germany) with shaking as described previously [34]. After digestion cells were filtered through a 70 μm mesh filter. The isolated cells were collected by centrifugation at 240 g for 10 min. Cell numbers of primary adipose cells were determined with a hemocytometer. Isolated equine ASCs were grown in monolayer culture as described earlier [35]. When cells had reached 80% of confluence, they were detached from the culture dish using TripleE (Invitrogen, Germany). The ASCs used for this study showed in earlier studies the potential of tri-lineage differentiation and were characterized by PCR and Flow Cytometry as described by Raabe et al. 2013 [35].

### Isolation of exosomes

Equine ASC were cultured in exosome-depleted medium which was obtained by ultracentrifugation of a DMEM low glucose medium (Thermo Fisher Scientific, Germany) in the presence of 5% FCS (Biosell, Germany) at 100.000 g over night at 4 °C. Near confluent cells (80–90%) were washed with 20 ml of DMEM low glucose without FCS on a shaker and were incubated in a humified incubator (37 °C in 5% CO_2_) in 10 ml DMEM low glucose with supplementation of 1x ITS (Insulin Transferrin-Selenium, Thermo Fisher Scientific) for 48–72 h. Supernatant was collected and centrifuged at 2.700 g for 5 min and afterwards filtered through a 0.2 μm filter (Sarstedt, Germany). Exosomes were isolated from this filtrate with three different isolation procedures.

### Isolation of exosomes by ultracentrifugation

For the standard isolation technique ultracentrifugation was applied. After filtration of the supernatants through a 0.2 μm filter as mentioned above the filtrate was subjected to different centrifugation steps at 4 °C, starting with a 10 min centrifugation step at 2.000 g, followed by a centrifugation at 10.000 g for 30 min. The supernatant was then further centrifuged by ultracentrifugation at 100.000 g (50.2 Ti Rotor Beckmann) for 70 min followed by another ultracentrifugation step of the in PBS resuspended pellet at 100.000 g for additional 70 min. The resultant pellet was washed with PBS, and after ultracentrifugation, exosomes were resuspended in lysis buffer.

### Isolation of exosomes by ultrafiltration

Another isolation step consisted of an ultrafiltration procedure which was carried out according to the size of the exosomes. For this procedure floating cells were centrifuged at 3.000 g for 5 min and the supernatant was also filtered through a 0.2 μm filter to remove cellular debris. 10–20 ml of this filtrate was subject to ultrafiltration using a 3 K Ultra Filter (Merck Milipore, Germany) and was centrifuged at 2.700 g until 100–200 μl are left. Afterwards the concentrate was washed in 4 ml PBS and again centrifuged at 2.700 g at 4 °C. This washing step was repeated three times. The supernatant was concentrated 100x and used for further experiments.

### Isolation of exosomes by precipitation

As a third method a charge-based precipitation was performed to obtain the exosomes. For this procedure supernatants with ASC derived exosomes were submitted to two centrifugation steps at 3.000 g for 20 min to remove cell debris and other contaminants. Afterwards the supernatants were filtered through a 0.2 μm filter. Then the samples were transferred in sterile vials and 15 μl 2% protaminesulfate (PS) and 300 μl 50% polyethylene glycol (PEG 35.000) were added. After incubation for 1 h at 4 °C the mixture was centrifuged at 12.000 g for 10 min at 4 °C and the supernatant was discarded. The pellet was re-suspended in the appropriate buffer for further experiments.

### Nanoparticle tracking analysis

Exosomes isolated from equine ASC were subjected to the Nanoparticle tracking analysis (NTA), using a NanoSight LM10 (Malvern Instruments Ltd., UK). The basic data about the processed particles that can be acquired by this method include average size, modal value and size distribution. These parameters were analyzed by the NTA 3.3 analytical software according to manufactures protocol. Settings were optimized and kept constant between samples. The Brownian movements of particles (exosomes) present in the sample were subjected to a laser beam and were recorded by a camera and converted into size and concentration parameters by NTA through the Stokes-Einstein equation. Every sample was measured in triplicate.

### Immunohistochemistry

The immunofluorescence staining was performed as previously described [35]. In brief equine stem cells were plated in a 96 well plate with a density of 10.000 cells per well. After 3 days when the cells had reached near confluency medium was aspirated from the cells and they were washed with PBS. After fixation with 4% paraformaldehyde for 20 min at room temperature MSCs were incubated over night with primary antibodies. The antibodies used in this study include the following: anti CD9 (monoclonal mouse, HI9a, BioLegend, 1:1000), anti CD63 (monoclonal mouse, MX-49.129.5, Santa Cruz, 1:500) and anti CD81 (monoclonal mouse, sc-166,029, Santa Cruz, 1:500). For negative controls the primary antibody was replaced by non-immune serum (rat anti mouse IgG isotypes, Invitrogen, 1:200). Then a secondary goat anti mouse Cy3 antibody (Dianova, Hamburg, Germany, 1:150) was applied for 1 h. Counterstaining was performed by using Hoechst-dye (bisBenzimide H33258, Sigma-Aldrich, Steinheim, Germany).

### Western blot

Exosomes were lysed, denatured in SDS buffer (0.4% SDS, 0.2 M Tris-HCl, pH 6.8, 5% glycerol, 0.02% bromophenol blue, 1% 2-mercaptoethanol) at 95 °C for 10 min. Proteins were obtained and resolved by 10% sodium dodecyl polyacrylamide gel electrophoresis (SDS-PAGE), and were then transferred to a nitrocellulose membrane (BioTrace NT, VWR, Darmstadt). The membranes were blocked for 2 h with 5% non-fat milk powder and afterwards incubated overnight at 4 °C with antibodies against CD9 (see above) and CD90 (monoclonal mouse, 5E10, BD, 1:1000). As secondary antibody a horseradish peroxidase (HRP)-conjugated goat anti-mouse IgG was applied for 1 h at ambient temperature. Luminescent visualization was done using an ECL kit (Merck Milipore, Darmstadt, Germany) to identify immunoreactive protein bands.

### Transmission electron microscopy (TEM) and immunogold labeling

Exosomes were fixed in 2% paraformaldehyde (PFA) solution and applied on Formvar-carbon coated grids (TAAB Laboratories). Samples were washed with PBS and fixed for 5 min with 1% glutaraldehyde. Fixated grids were washed with deionized water and subsequently contrasted for 5 min with an uranyl-oxalate solution (4% uranyl acetate, 0.15 M oxalic acid, pH 7, Sigma-Aldrich). At the end, grids were air dried for 10 min and examined under a Zeiss EM 109 with an accelerating voltage of 80 kV. For immunogold staining, 5 μl of resuspended paraformaldehyde fixed exosomes were transferred on glow discharged Formvar-carbon coated nickel grids. After washing with PBS, the grids were incubated for 3 min with 50 mM glycine/PBS. The specimens on the grids were permeabilized with 0.1% saponin for membrane protein labeling before they were washed and blocked with 0.5% BSA. Exosomes were incubated with the following monoclonal primary antibodies: mouse anti CD9 (clone HI9a, antibodies-online, 1:10), mouse anti CD81 (sc-166,029, Santa Cruz, 1:20) and mouse anti CD90 (clone 5E10, BD, 1:40), washed and labeled with a goat anti mouse secondary antibody conjugated to 10 nm gold particles (EM.GMHL10, BBI Solutions, 1:15). Grids were contrasted as described above. Observations were carried out with a Zeiss EM 109 with an accelerating voltage of 80 kV (Zeiss Oberkochen, Germany).

## Abbreviations

ASC: adipose derived stem cells
FCS: fetal calf serum
MSC: mesenchymal stem cells
NTA: nanoparticle tracking analysis
PEG: polyethylene glycol
TEM: transmission electron microscopy
